# Burden of Disease, Injuries, and Risk Factors in the Kingdom of Saudi Arabia, 1990–2010

**DOI:** 10.5888/pcd11.140176

**Published:** 2014-10-02

**Authors:** Ziad A. Memish, Sara Jaber, Ali H. Mokdad, Mohammad A. AlMazroa, Christopher J.L. Murray, Abdullah A. Al Rabeeah, the Saudi Burden of Disease Collaborators

**Affiliations:** Author Affiliations: Sara Jaber, Ali H. Mokdad, Christopher J.L. Murray, Institute for Health Metrics and Evaluation, University of Washington, Seattle, Washington, USA; Mohammad A. AlMazroa, Abdullah A. Al Rabeeah, Ministry of Health of the Kingdom of Saudi Arabia, Assadah, Al Murabba Riyadh, Saudi Arabia.

## Abstract

**Introduction:**

We report the burden of disease and risk factors measured by causes of death, years of life lost attributable to premature mortality (YLLs), years of life lived with disability (YLDs), and disability-adjusted life years (DALYs) for 1990, 2005, and 2010 in the Kingdom of Saudi Arabia (KSA).

**Methods:**

We used the Global Burden of Diseases 2010 (GBD 2010) methodology to estimate the country-level burden of disease in KSA. We used data from systematic reviews of the literature, household survey data, antenatal clinic surveillance data, reportable disease notifications, disease registries, hospital admissions data, outpatient visit data, population-based cancer registries, active screening data, and other administrative data.

**Results:**

Noncommunicable diseases and road traffic injuries became the leading cause of death and disability in KSA in 2010. Elevated body mass index was the leading risk factor for disease (7.02% for males and 4.61% for females in 2010). High glucose levels were the second leading disease risk factor for females (3.28%) and third for males (6.25%) in 2010. Preterm birth complications were the main cause for DALYs in 1990; however, in 2010, the leading cause of DALYs for males was road traffic injuries (12.40%) and for females it was major depressive disorder (7.88%).

**Conclusion:**

KSA is facing a rising burden of noncommunicable diseases and road traffic injuries as a result of rapid changes in behaviors. Our results demonstrate the need for major intervention to reduce these burdens and to engage other sectors of the government and the community in these efforts.

## Introduction

The Kingdom of Saudi Arabia (KSA) made tremendous improvements in its health care systems in a short time because of extensive investments (1,2). The Ministry of Health (MOH) is responsible for 60% of the health care services, with the remaining 40% managed by numerous semipublic organizations and the private sector (3). In 2010, the MOH employed 250,000 personnel (including 31,516 physicians and 75,978 nurses), and it operated 249 hospitals with 34,000 beds (4).

KSA faces several health challenges that are unusual for a country with high income. The religious sites are visited by millions of pilgrims throughout the year, and a mass gathering occurs during Hajj (5). Although there are strict requirements for vaccination to avoid disease outbreaks during Hajj, the sheer number of visitors contributes to increased infectious disease burden (5). Moreover, illegal migration to KSA for work or Hajj adds to this burden because most illegal immigrants do not have the required vaccinations. In recent years and with improvements in infrastructure and health services, an emerging burden of noncommunicable diseases (NCDs) is unfolding; studies found high rates of obesity, diabetes, and high blood pressure (6–10). Moreover, with increased use of motor vehicles, the burden of road traffic injuries increased (11).

The MOH is investing in reforming its health information systems and in May 2012 began a collaboration with the Institute for Health Metrics and Evaluation (IHME) to implement an integrated health information system within the next 5 years. The collaboration involves creating a database of the burden of disease, injuries, and risk factors for KSA at the national and local level. In this article, we report the national burden of disease in KSA for 1990 through 2010 based on the results of the Global Burden of Disease 2010 (GBD 2010) project (12).

## Methods

The GBD 2010 was a systematic scientific effort to quantify the comparative magnitude of loss of health for 187 countries from 1990 to 2010. The GBD 2010 covered 291 diseases and injuries, 1,160 sequelae of these diseases and injuries, and 67 risk factors or clusters of risk factors for various diseases or injuries (12–18). GBD 2010 estimates the burden of disease and injury by age, sex, and country for 1990, 2005, and 2010 (8–14). Loss of health was assessed on the basis of a systemic analysis of all the available data by using the following metrics: mortality, causes of death, years of life lost attributable to premature mortality (YLLs), years of life lived with disability (YLDs), and disability-adjusted life years (DALYs).

DALYs provide a summary measure of premature mortality and time spent in less than ideal health (12). YLLs, YLDs, and DALYs measure loss of health in terms of time (12). DALYs are the sum of YLLs and YLDs (12). YLLs are the number of deaths attributed to a disease multiplied by the standard life expectancy at the age of death in years (12). YLD is the prevalence of each disease or injury sequela multiplied by the associated disability weight for that sequela and the duration until the person with the disease dies or the disease goes into remission (12).

### Mortality

A detailed description of how age-specific mortality was estimated for each sex, country, and year is published elsewhere (15). Data on mortality come from various sources depending on the availability of data. For countries with abundant resources, information on deaths is from official vital registration systems (15). In low- and middle-income countries, multiple sources of data may be used in an attempt to achieve as complete all-cause mortality estimates as possible (12–18).

### Risk factors

Sixty-seven risk factors or clusters of risk factors responsible for mortality, YLLs, YLDs, or DALYs were examined in GBD 2010. The attributable deaths or DALYs associated with each risk factor were assessed by using 4 components: 1) a database on risk factor–exposure from the published literature, 2) estimates of the prevalence of risk factor–exposure by country, age, and sex based on both published and unpublished sources using mostly Bayesian methods, 3) estimates of the relative risks for risk–disease pairs based on published and unpublished data, and 4) comparison of the current distribution of exposure to a counterfactual distribution called the theoretical minimum risk distribution (13) for each risk factor. Each risk factor or risk factor cluster was analyzed separately, such that the sum of attributable fractions for a disease or injury may be greater than 100%. Uncertainty in the relative risks, exposure estimates, theoretical minimum risk distributions, and in the background outcome rates were considered in the final estimates.

IHME in collaboration with the KSA MOH developed a database of published and unpublished data sources to estimate the burden of disease for KSA. Key sources for this database included data from systematic reviews of the literature, household surveys, antenatal clinic surveillance, reportable disease notifications, disease registries, hospital admissions, outpatient visits, population-based cancer registries, screening results, and other administrative sources. These data sources were used to estimate the disease and disability burden for 1990, 2005, and 2010.

## Results

**Figure 1:**
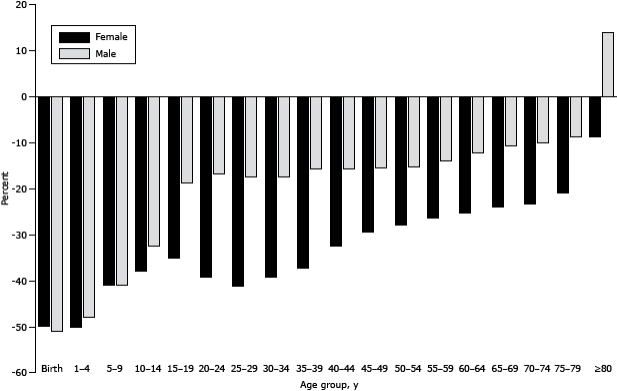
Percentage decline in mortality from 1990 to 2010, by sex, Kingdom of Saudi Arabia.

**Figure 2:**
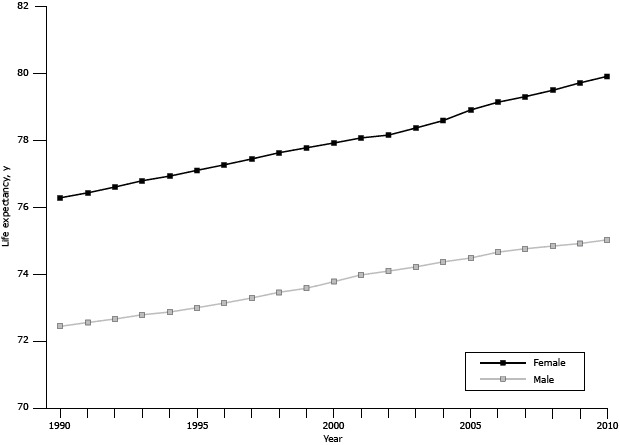
Life expectancy at birth by sex, Kingdom of Saudi Arabia, 1990–2010.

From 1990 through 2010, there was a decline in age-specific mortality in KSA (Figure 1). The greatest reductions in all-cause mortality were among males aged less than 1 year (51%). Men aged 80 years or older had an increase in mortality (14%). During the same time, life expectancy increased from 72.5 to 75.0 for men and from 76.3 to 79.9 for women (Figure 2). During the same time, healthy life expectancy increased from 61.8 to 63.9 for men and from 63.5 to 66.6 for women. Infant mortality declined from 23.8 per 1,000 to 11.9 per 1,000, and maternal mortality ratios declined from 30.0 to 15.0 per 100,000 live births.

The main cause of death in 1990 was ischemic heart disease (IHD), accounting for 16.04% of total mortality (Table 1). Road traffic injuries were the third leading cause of death in 2010, accounting for 11.75% of total mortality. The patterns in causes of death were different for males and females throughout the study period.

**Table 1:** Leading Causes of Death by Sex, Kingdom of Saudi Arabia, 1990, 2005, and 2010

Rank	1990			2005			2010		
	Male (%)	Female (%)	Total (%)	Male (%)	Female (%)	Total (%)	Male (%)	Female (%)	Total (%)
1	Ischemic heart disease (17.19%)	Cerebrovascular disease (14.24%)	Ischemic heart disease (16.04%)	Ischemic heart disease (19.56%)	Ischemic heart disease (16.84%)	Ischemic heart disease (18.65%)	Ischemic heart disease (19.25%)	Ischemic heart disease (17.94%)	Ischemic heart disease (18.77%)
2	Road traffic injury (13.64%)	Ischemic heart disease (14.20%)	Cerebrovascular disease (11.39%)	Road traffic injury (15.08)	Cerebrovascular disease (15.35%)	Cerebrovascular disease (12.81%)	Road traffic injury (16.08%)	Cerebrovascular disease (16.93%)	Cerebrovascular disease (13.20%)
3	Cerebrovascular disease (9.62%)	Preterm birth complications (11.24%)	Preterm birth complications (10.12%)	Cerebrovascular disease (11.54%)	Preterm birth complications (6.91%)	Road traffic injury (11.71%)	Cerebrovascular disease (11.05%)	Lower respiratory infections (5.97%)	Road traffic injury (11.75%)
4	Preterm birth complications (9.43%)	Congenital anomalies (6.89%)	Road traffic injury (9.99%)	Lower respiratory infections (5.12%)	Lower respiratory infections (5.39%)	Preterm birth complications (5.24%)	Lower respiratory infections (5.01%)	Preterm birth complications (5.05%)	Lower respiratory infections (5.36%)
5	Lower respiratory infections (5.73%)	Lower respiratory infections (6.52%)	Lower respiratory infections (6.03%)	Preterm birth complications (4.40%)	Road traffic injury (5.02%)	Lower respiratory infections (5.21%)	Chronic kidney diseases (3.90%)	Road traffic injury (4.22%)	Preterm birth complications (4.11%)
6	Congenital anomalies (4.46%)	Road traffic Injury (4.11%)	Congenital anomalies (5.39%)	Chronic kidney diseases (3.50%)	Congenital anomalies (4.41%)	Chronic kidney diseases (3.52%)	Diabetes mellitus (3.65%)	Chronic kidney diseases (4.15%)	Chronic kidney diseases (3.99%)
7	Hypertensive heart disease (1.90%)	Diarrheal diseases (2.82%)	Diarrheal diseases (2.22%)	Diabetes mellitus (3.24%)	Chronic kidney diseases (3.55%)	Diabetes mellitus (3.27%)	Preterm birth complications (3.56%)	Diabetes mellitus (3.79%)	Diabetes mellitus (3.70%)
8	Neonatal encephalopathy (1.90%)	Neonatal encephalopathy (2.32%)	Neonatal encephalopathy (2.06%)	Hypertensive heart disease (2.42%)	Diabetes mellitus (3.34%)	Congenital anomalies (3.06%)	Hypertensive heart disease (2.37%)	Congenital anomalies (3.04%)	Hypertensive heart disease (2.37%)
9	Diarrheal diseases (1.84%)	Sepsis and other infectious disorders of newborn (1.97%)	Hypertensive heart disease (1.88%)	Congenital anomalies (2.39%)	Other cardiovascular and circulatory diseases (2.40%)	Hypertensive heart disease (2.37%)	Congenital anomalies (1.91%)	Other cardiovascular and circulatory diseases (2.60%)	Congenital anomalies (2.32%)
10	Chronic obstructive pulmonary disease (1.75%)	Other cardiovascular and circulatory diseases (1.95%)	Chronic obstructive pulmonary disease (1.73%)	Other cardiovascular and circulatory diseases (1.69%)	Hypertensive heart disease (2.28%)	Other cardiovascular and circulatory diseases (1.93%)	Other cardiovascular and circulatory diseases (1.64%)	Hypertensive heart disease (2.38%)	Other cardiovascular and circulatory diseases (1.99%)

**Figure 3:**
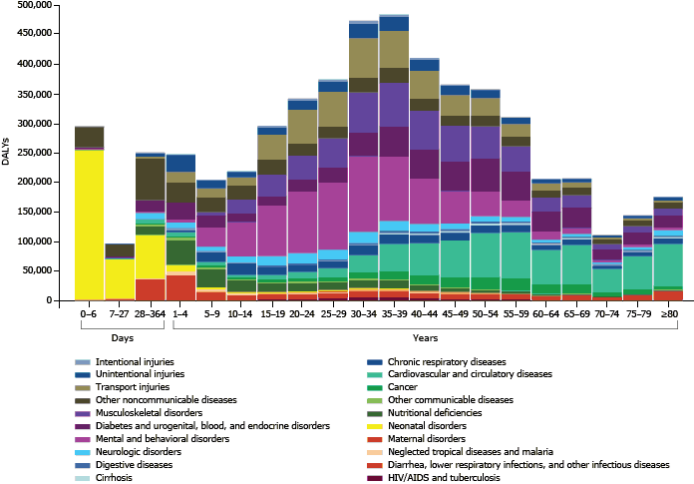
Disability-adjusted life years (DALYs) by cause and age, Kingdom of Saudi Arabia, 2010.

In 2010, the main causes of DALYS in KSA were major depressive disorder, road traffic injuries, IHD, and diabetes (Table 2). This change in leading causes is a major shift from preterm birth complications in 1990 (12.17%). Road traffic injuries were mostly concentrated in age groups from 15 through 54 years (Figure 3). Mental disorders were concentrated mostly in age groups from 15 through 44 years.

**Table 2:** Leading Causes of Disability-Adjusted Life Years by Sex, Kingdom of Saudi Arabia, 1990, 2005, and 2010

Rank	1990			2005			2010		
	Male (%)	Female (%)	Total (%)	Male (%)	Female (%)	Total (%)	Male (%)	Female (%)	Total (%)
1	Preterm birth complications (12.04%)	Preterm birth complications (12.35%)	Preterm birth complications (12.17%)	Road traffic injury (12.00%)	Major depressive disorder (7.56%)	Road traffic injury (8.55%)	Road traffic injury (12.40%)	Major depressive disorder (7.88%)	Road traffic injury (8.63%)
2	Road traffic injury (11.04%)	Congenital anomalies (7.65%)	Road traffic injury (7.79%)	Ischemic heart disease (7.38%)	Preterm birth complications (6.94%)	Preterm birth complications (6.38%)	Ischemic heart disease (7.46%)	Preterm birth complications (5.58%)	Ischemic heart disease (6.23%)
3	Ischemic heart disease (6.03%)	Major depressive disorder (5.72%)	Congenital anomalies (6.53%)	Preterm birth complications (6.00%)	Diabetes mellitus (4.71%)	Ischemic heart disease (6.12%)	Diabetes mellitus (5.79%)	Diabetes mellitus (5.10%)	Major depressive disorder (5.87%)
4	Congenital anomalies (5.71%)	Iron-deficiency anemia (3.86%)	Ischemic heart disease (5.09%)	Low back pain (5.37%)	Low back pain (4.64%)	Major depressive disorder (5.61%)	Low back pain (5.77%)	Low back pain (5.06%)	Diabetes mellitus (5.51%)
5	Low back pain (3.88%)	Ischemic heart disease (3.78%)	Major depressive disorder (4.43%)	Diabetes mellitus (5.26%)	Congenital anomalies (4.54%)	Low back pain (5.08%)	Preterm birth complications (4.79%)	Ischemic heart disease (4.46%)	Low back pain (5.48%)
6	Major depressive disorder (3.51%)	Lower respiratory infections (3.69%)	Low back pain (3.51%)	Major depressive disorder (4.29%)	Ischemic heart disease (4.26%)	Diabetes mellitus (5.04%)	Major depressive disorder (4.47%)	Anxiety disorders (4.30%)	Preterm birth complications (5.11%)
7	Lower respiratory infections (2.94%)	Diarrheal diseases (3.41%)	Lower respiratory infections (3.26%)	Cerebrovascular disease (3.54%)	Anxiety disorders (4.10%)	Congenital anomalies (3.79%)	Cerebrovascular disease (3.46%)	Iron deficiency anemia (3.75%)	Cerebrovascular disease (3.44%)
8	Cerebrovascular disease (2.77%)	Road traffic injury (3.28%)	Iron deficiency anemia (3.09%)	Congenital anomalies (3.28%)	Iron deficiency anemia (3.89%)	Cerebrovascular disease 3.41%)	Drug-use disorders (2.75%)	Congenital anomalies (3.48%)	Congenital anomalies (2.97%)
9	Neonatal encephalopathy (2.62%)	Cerebrovascular disease (3.27%)	Cerebrovascular disease (2.98%)	Drug-use disorders (2.31%)	Road traffic injury (3.48%)	Iron deficiency anemia (2.81%)	Congenital anomalies (2.62%)	Cerebrovascular disease (3.41%)	Anxiety disorders (2.88%)
10	Diabetes mellitus (2.62%)	Low back pain (3.00%)	Diarrheal diseases (2.86%)	Iron deficiency anemia (2.08%)	Cerebrovascular disease (3.22%)	Anxiety disorders (2.77%)	Iron deficiency anemia (1.92%)	Road traffic injury (3.20%)	Iron deficiency anemia (2.67%)

Metabolic risk factors are the main causes of DALYs in KSA for 1990, 2005, and 2010 (Table 3). For 2005 and 2010, elevated body mass index (BMI) and dietary risks continued to be the leading disease risk factors (for BMI, 10.20% in 2005 and 11.64% in 2010; for dietary risks, 9.25% in 2005 and 9.68% in 2010).

**Table 3:** Leading Risk Factors Attributable to Disability-Adjusted Life Years by Sex, Kingdom of Saudi Arabia, 1990, 2005, and 2010

Rank	1990			2005			2010		
	Male (%)	Female (%)	Total (%)	Male (%)	Female (%)	Total (%)	Male (%)	Female (%)	Total (%)
1	Dietary risks (4.49%)	Dietary risks (2.27%)	Dietary risks (6.76%)	Dietary risks (6.44%)	Elevated BMI (3.98%)	Elevated BMI (10.20%)	Elevated BMI (7.02%)	Elevated BMI (4.61%)	Elevated BMI (11.64%)
2	Elevated blood pressure (3.64%)	Elevated blood pressure (2.27%)	Elevated blood pressure (5.91%)	Elevated BMI (6.22%)	Elevated FPG (2.90%)	Dietary risks (9.25%)	Dietary risks (6.64%)	Elevated FPG (3.28%)	Dietary risks (9.68%)
3	Elevated BMI (2.96%)	Elevated BMI (2.17%)	Elevated BMI (5.13%)	Elevated FPG (5.63%)	Dietary risks (2.81%)	Elevated FPG (8.53%)	Elevated FPG (6.25%)	Dietary risks (3.04%)	Elevated FPG (9.53%)
4	Elevated FPG (2.59%)	Iron deficiency anemia (1.67%)	Elevated FPG (4.05%)	Elevated blood pressure (4.99%)	Elevated blood pressure (2.47%)	Elevated blood pressure (7.46%)	Elevated blood pressure (5.19%)	Elevated blood pressure (2.64%)	Elevated blood pressure (7.83%)
5	Ambient air pollution (2.36%)	Elevated FPG (1.46%)	Ambient air pollution (3.78%)	Physical inactivity (3.32%)	Physical inactivity (1.66%)	Physical inactivity (4.98%)	Physical inactivity (3.51%)	Physical inactivity (1.82%)	Physical inactivity (5.33%)
6	Smoking (1.65%)	Ambient air pollution (1.42%)	Iron deficiency anemia (3.14%)	Ambient air pollution (2.77%)	Iron deficiency anemia (1.59%)	Ambient air pollution (4.02%)	Ambient air pollution (2.77%)	Iron deficiency anemia (1.55%)	Ambient air pollution (4.05%)
7	Occupational risks (1.61%)	Suboptimal breastfeeding (0.95%)	Smoking (2.40%)	Smoking (2.11%)	Ambient air pollution (1.25%)	Iron deficiency anemia (2.82%)	Smoking (2.49%)	Ambient air pollution (1.27%)	Smoking (3.02%)
8	Iron deficiency anemia (1.47%)	Household air pollution (0.92%)	Household air pollution (2.23%)	Occupational risks (1.88%)	Elevated total cholesterol (0.72%)	Smoking (2.79%)	Occupational risks (1.91%)	Intimate partner violence (0.73%)	Iron deficiency anemia (2.68%)
9	Elevated total cholesterol (1.47%)	Childhood underweight (0.87%)	Elevated total cholesterol (2.23%)	Elevated total cholesterol (1.49%)	Smoking (0.68%)	Elevated total cholesterol (2.21%)	Drug-use disorders (1.66%)	Elevated total cholesterol (0.66%)	Drug-use disorders (2.24%)
10	Household pollution (1.31%)	Elevated total cholesterol (0.76%)	Suboptimal breastfeeding (1.83%)	Drug-use disorders (1.41%)	Intimate partner violence (0.65%)	Occupational risks (2.01%)	Elevated total cholesterol (1.32%)	Drug-use disorders (0.58%)	Occupational risks (2.05%)

Abbreviations: BMI, body mass index; FPG, fasting plasma glucose.

In 2010, DALYs from NCDs were highest among older age groups and started increasing after age 40 years (Figure 3). Mental and behavioral disorders were highest among those aged 20 through 39 years. Musculoskeletal disorders were high among those aged 30 through 44 years.

## Discussion

Our study shows that KSA is facing a rising burden of road traffic accidents (RTAs), major depressive disorder, and NCDs. These findings are a clear indication of KSA’s success in controlling infectious diseases and reducing their prevalence. The rise of RTAs and NCDs are a clear warning of the need for immediate intervention. The major challenge ahead for KSA is reducing the burden of chronic diseases and their risk factors. As the country’s population ages and grows, NCDs will pose a major challenge to KSA even with its wealth and resources.

Our findings on the huge burden of NCDs in KSA are similar to those we observed in the GBD 2010 results for other Gulf countries. NCDs are becoming a major burden in the region and in the Arab world (19). The rapid change in the burden profile in the region is alarming. Diabetes, obesity, high blood pressure, cancers, and cardiovascular diseases are putting a huge toll on the health care systems and on society. This transition needs a planned intervention to control and prevent the future burden of disease. Most governments in the region have not given attention to NCDs as they have given or are giving to infectious disease. A new culture of health care in KSA and the region should be a priority.

Our findings on DALYs in KSA deserve special attention. First, there are huge variations in the burden by age. Second, it is clear that mental health is becoming a major challenge for KSA. This could be due to societal changes and a move away from tradition where family support was always available. Third, it is clear that what is killing Saudis is different from what is ailing them: mental and musculoskeletal disorders are becoming a major challenge for the Saudi health system but not a major cause of death. Finally, NCDs are the main challenge because they are a major cause of disability and death.

Despite the growth of NCDs and RTAs, KSA is a unique country where outbreaks of infectious disease are always a threat because of the large number of pilgrims visiting the Kingdom throughout each year and especially during the Hajj season. This threat requires lots of effort and resources for infectious disease control and prevention. Conditions surrounding the Hajj such as extended stays in a single geographic area, physical exhaustion, extreme heat, and crowded accommodations usually lead to disease transmission, especially of airborne agents (5). The novel beta coronavirus called HCoV-EMC that infected a Saudi patient in June 2012 is a reminder that outbreaks will continue to be a major challenge (20).

The changing patterns of disease in KSA have forced the MOH to adapt its mission and operation to deliver timely solutions for emerging challenges. These challenges include issues of workforce development, financing, insurance, accessibility, and use of electronic health resources (1). The MOH implemented several new programs to control and prevent disease. A compulsory vaccination program, which started in the 1980s and has been updated since, has led to a substantial drop in mortality among those aged less than 5 years, from 250 per 1,000 live births in 1960 to 26 per 1,000 live births in 2005 (21). An innovative 5-year project was launched in 2012 in collaboration with IHME to create an integrated tracking system to monitor the health status of Saudi citizens and determine health policy priorities. The project includes determining the burden of disease and risk factors at the local level.

The changes in lifestyle habits because of the recent economic growth are troubling. The prevalence of physical activity is low in the Kingdom (22). The hot weather and increased urbanization are not ideal for encouraging or promoting physical activities. Moreover, poor dietary behaviors are common in KSA (23). Obesity is a major problem in the country with a prevalence of 42.4% among males and 31.8% among females (24). As a result, KSA has a high prevalence of metabolic syndrome of 39.3% (25) and coronary artery diseases of 5.5% (26). Smoking prevalence in KSA ranges from 1.4% among females aged 15 through 64 years to 24.2% among males aged 15 through 64 years (27,28). The prevalence differs among the age groups, with males more likely than females to smoke (median for males, 26.5%, and for females, 9%) (27). Another important issue is water-pipe smoking (known as shisha), which seems to be highly prevalent among teenagers (29).

In September 2012, the MOH in KSA in collaboration with the World Health Organization (WHO) regional office for the Eastern Mediterranean (EMRO) organized an international conference to address the topic of NCDs in the area (30). The conference released the Riyadh Declaration that included 10 recommendations to combat NCDs at the regional level (Appendix). The MOH has worked with WHO/EMRO, and the declaration was adopted by EMRO during the regional committee meeting in October 2012. These recommendations will have a major impact on health in KSA and the region.

RTAs are now a major burden in KSA because of nonadherence to traffic and safety laws. The patterns of driving and traffic regulations changed from 1990 to 2010. RTAs have a major impact on the working population in terms of lost productivity (11). The MOH in collaboration with the Ministry of Interior launched a road safety program called Saher in 2009 (31). Saher is an automated system that was developed to manage traffic via electronic systems in major cities in Saudi Arabia (31). This newly established system uses a digital camera network connected to the National Information Center to track any violations and to control traffic (31). However, data on its long-term effects on accidents and deaths are not yet available. Other efforts to reduce RTAs should focus on police enforcement of traffic laws. RTAs would be much lower if the police were to ensure that drivers have a license and that all vehicle occupants are wearing seat belts.

Societal mores play an important role in health promotion. Individual well-being is directly related to the strength of the social relationships and support that a person receives (32). The problems that the Saudi health system is facing require multi-sectorial interventions with roles and responsibilities for many players. Perhaps an important role should be played by the religious community. Religious institutions can be a source of social support by stressing values that influence health-related behavior (33). Faith organizations can influence the health education, health promotion, and positive health outcomes of the members of their faith community (33).

KSA is a country with deep cultural roots and traditions, including involvement in sports and physical activity. KSA needs role models who can encourage people to improve their diet and increase their level of physical activity.

Our study has limitations. We used the most recent data on the prevalence of NCD risk factors from the 2005 WHO STEPwise Approach to NCD Surveillance and the 2008 World Health Survey (28,34) to report the burden in 2010. It is possible that the prevalence has changed since then. In addition, mortality data in KSA may be underreported especially for women. However, we applied our standard techniques to deal with garbage coding and underreporting to produce our estimates (18,19). Moreover, we incorporated a wealth of information and data into our work.

Our findings demonstrate the need for developing and implementing programs to reduce health risk factors in KSA. Such programs need to be locally developed and adopted because many programs described in the literature may not succeed in KSA. Programs to promote physical activity especially among women need to be culturally sensitive and appropriate for the harsh weather in KSA. Many programs will require political will and legislation to succeed. Perhaps gyms could be built where physical activity programs would be available and supervised by medical staff. Moreover, police must enforce driving laws, especially those related to speed, safety, and driver’s age. Finally, religious and community leaders should have a role in in all mass campaigns to improve health.

## Appendix. Recommendations from the Riyadh Declaration

Following deliberations and debates, experts gathered at the International Conference on Healthy Lifestyles and Non-communicable Diseases (NCDs) in the Arab World and the Middle East, held in Riyadh, Kingdom of Saudi Arabia, in September 2012, recommended the following immediate action points:

1. An annual screening package for early components of the metabolic syndrome (pre-hypertension, pre-diabetes, overweight, tobacco addiction) should be available to all asymptomatic adults from age 25 years, through primary health care facilities, fully or largely subsidized based on the health insurance system and available finances in each country.
2. Individuals diagnosed through the screening package should be referred to adequate and accessible care.
3. Schools must be recognized as a major venue for NCD prevention. Accreditation or rehabilitation of educational facilities for boys and girls should be based on the criteria of World Health Organization Health Promoting Schools. In particular, physical education and access to healthy food items should be considered as priorities in the educational system, equal in importance to reading and writing.
4. Urban planning licenses of new residential developments have to include environments that promote walking or biking, social gathering, and safe space to allow physical activity for women, elderly persons, and children.
5. Adopt the mandatory use of traffic light signs on all industrial food items imported or locally manufactured.
6. Impose nutritional labeling on all fast food items.
7. Impose the sale of fresh fruits and vegetables as well as low-calorie products in all vending venues where high-calorie equivalents are sold.
8. Require a gradual reduction over the coming 5 years of the salt content of all manufactured food items, to ultimately reach 50% of the initial content.
9. Ban all shisha smoking cafes from residential areas and neighborhoods with health or educational facilities.
10. Increase the taxation on items with negative health effects (tobacco products, energy drinks), and earmark obtained funds to NCD programs.
